# Iridium-catalyzed intramolecular [4 + 2] cycloadditions of alkynyl halides

**DOI:** 10.3762/bjoc.8.201

**Published:** 2012-10-16

**Authors:** Andrew Tigchelaar, William Tam

**Affiliations:** 1 Guelph-Waterloo Centre for Graduate Work in Chemistry and Biochemistry, Department of Chemistry, University of Guelph, Guelph, Ontario, Canada N1G 2W1

**Keywords:** alkynyl halide, cycloaddition, diene-tethered alkyne, iridium, transition-metal catalyst

## Abstract

Iridium-catalyzed intramolecular [4 + 2] cycloadditions of diene-tethered alkynyl halides were investigated by using [IrCl(cod)]_2_ as catalyst, and dppe was found to be the most suitable phosphine ligand for the reaction. No oxidative insertion of the iridium into the carbon–halide bond was observed, and the reactions proceeded to provide the halogenated cycloadducts in good yield (75–94%). These results are the first examples of cycloadditions of alkynyl halides using an iridium catalyst.

## Introduction

Iridium complexes have been used as catalysts for a wide variety of reactions, including homogeneous hydrogenation [1–2], C–H activation [3–5], asymmetric ring-opening reactions [6], and a variety of cycloisomerizations [7–10], and cycloadditions [11–20]. Traditionally these types of cycloisomerization and cycloaddition reactions are possible by making use of other metal complexes such as Pd [21–22] and Co [23–25], but recent advances in iridium chemistry have expanded the scope of the metal complexes that can be used. Common Ir-catalyzed cycloadditions include [2 + 2 + 2] cycloadditions of diynes [11–13] or enynes [16] with alkynes, [2 + 2 + 2] cyclotrimerizations of alkynes [14–15], and Pauson–Khand-type [2 + 2 + 1] cycloadditions of enynes [17–19] and allenynes [20]; however, Ir-catalyzed [4 + 2] cycloadditions are rare in the literature [26–27].

**Scheme 1:**
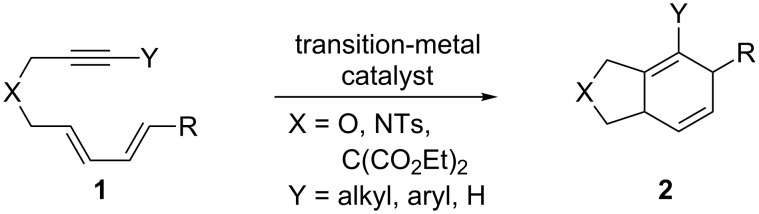
Literature examples of intramolecular TMC [4 + 2] cycloadditions of diene-tethered alkynes.

Transition-metal-catalyzed (TMC) [4 + 2] cycloadditions are an efficient way of making 6-membered rings, particularly in the case of electronically similar dienes and dienophiles, which can require high temperatures and long reaction times for the thermal cycloaddition to occur [28]. Many different transition-metal catalysts have been described for these types of reactions, employing a variety of different metals including Ni [29], Co [30], Rh [31–34], Pd [35–36] and Ir [26–27]. Much research has been done on intramolecular TMC [4 + 2] cycloadditions of a variety of diene-tethered alkynes to form substituted bicyclic products **2** (Scheme 1); however, the majority of these examples demonstrate Y = alkyl or aryl, and R = methyl. An interesting variation on this reaction is to utilize diene-tethered alkynyl halides (Y = halogen) in order to form a halogenated bicyclic product.

**Scheme 2:**
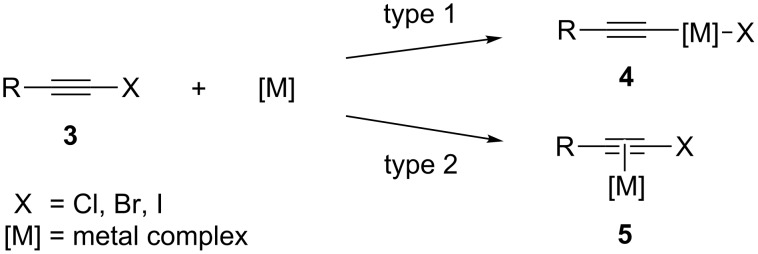
Reaction pathways of alkynyl halides in transition-metal-catalyzed reactions.

Alkynyl halides are a versatile moiety in organic synthesis, and can be prepared under mild conditions [37]. They can be seen as a dual functionalized molecule in TMC reactions, with the two main modes of reactivity proceeding by insertion of a metal into the carbon–halide bond (Scheme 2, type 1), or coordination of the π-system of the acetylene to the metal in a η^2^ fashion (Scheme 2, type 2). The most extensive studies done on alkynyl halides in TMC reactions are on cross-coupling reactions that proceed by oxidative insertion of the metal into the carbon–halide bond (type 1), and these types of reactions have been used to synthesize building blocks, such as enynes [38–39], diynes [40–41], and triynes [42–44]. Conversely, formation of intermediate **5** is rare, likely due to competition with oxidative insertion of the metal into the carbon–halide bond; however, there are reported cases of Co-catalyzed [2 + 2 + 1] [45], Ru-catalyzed [2 + 2] [46], and Rh-catalyzed [4 + 2] [33] cycloadditions that proceed via intermediate **5**.

## Results and Discussion

To the best of our knowledge, there are no known Ir-catalyzed [4 + 2] cycloadditions involving alkynyl halides. In this article, we report our studies on [IrCl(cod)]_2_/dppe-catalyzed intramolecular [4 + 2] cycloadditions of diene-tethered alkynyl halides. In order to begin the study, alkynyl bromide **1a** was synthesized as previously described [33], and several different catalytic iridium systems were screened (Table 1).

**Table 1:** Optimization of iridium-catalyst for [4 + 2] cycloaddition of alkynyl bromide **1a**.

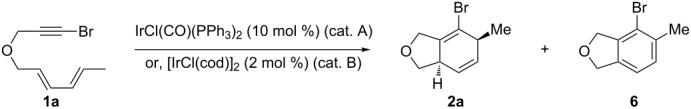							

Entry	Catalyst	Additive/ligand^a^ (4 mol %)^b^	Solvent	Temp. (°C)	Time (h)	Yield (%)^c^	
						**2a**	**6**

1	A	none	toluene	90	18	35	12
2	A	none	toluene	90	3	60	26
3	B	AgSbF_6_	acetone	25	1	0^d^	0
4	B	AgSbF_6_	toluene	90	3	0^d^	0
5	B	dppm	toluene	90	3	25	0
6	B	dppe	toluene	90	3	69	0
7	B	dppp	toluene	90	3	50	3
8	B	dppe	toluene	25	3	0^e^	0
9	B	PPh_3_	toluene	90	3	23	17
10	B	BINAP	toluene	90	3	28	18

^a^Abbreviations: dppm = 1,1-bis(diphenylphosphino)methane, dppe = 1,2-bis(diphenylphosphino)ethane, dppp = 1,3-bis(diphenylphosphino)propane, BINAP = 2,2'-bis(diphenylphosphino)-1,1'-binaphthyl. ^b^Except for entry 9, where 8 mol % was used for the mono-phosphine ligand. ^c^Isolated yields after column chromatography. ^d^Decomposition of starting material was observed. ^e^Only starting material was recovered.

Initial attempts were undertaken by using the catalyst system described by Shibata [26] employing Vaska’s complex in toluene (Table 1, entries 1 and 2) and cycloadduct **2a** was formed in good yield; however, the formation of the aromatized product **6** was also observed. Addition of the silver(I) salt AgSbF_6_ to the commercially available [IrCl(cod)]_2_ was attempted in order to generate the cationic Ir(I) species (Table 1, entries 3 and 4), but this resulted in decomposition of the starting material, both at room temperature and at 90 °C. A variety of phosphine ligands were screened in conjunction with [IrCl(cod)]_2_ [16,26], and these provided low to moderate yields of **2a**, with little to no formation of the aromatized cycloadduct **6** (Table 1, entries 5–8). Using racemic BINAP and PPh_3_ ligands also provided **2** in low yield, but a significant amount of **6** was also formed. From these observations, [IrCl(cod)]_2_ (2 mol %) and dppe (4 mol %) were carried forward, and a variety of solvents were screened at different temperatures in an attempt to further optimize the cycloaddition for cycloadduct **2a** (Table 2).

**Table 2:** Optimization of solvent and temperature for the [IrCl(cod)]_2_/dppe-catalyzed [4 + 2] cycloaddition of **1a**.

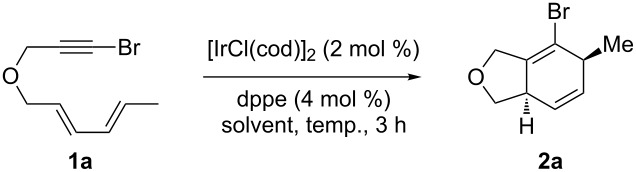			

Entry	Solvent	Temp. (°C)	Yield (%)^a^

1	toluene	90	69
2	1,4-dioxane	90	61^b^
3	DCE	75	70
4	acetone	45	0^c^
5	NMP	90	74
6	NMP	75	46^c^
7	DMSO	90	94
8	DMSO	65	25^c^
9	DMF	90	87

^a^Isolated yields after column chromatography. ^b^9% of aromatized cycloadduct **6** also recovered. ^c^Starting material recovered.

Employing nonpolar solvents 1,4-dioxane and 1,2-dichloroethane (Table 2, entries 2 and 3) provided very similar results to toluene, and the lower-boiling-point solvent acetone (Table 2, entry 4) was ineffective when heated to 45 °C. High-boiling-point, polar aprotic solvents NMP, DMSO, and DMF (Table 2, entries 5, 7, 9) all improved the yield of **2a** when the reaction was run at 90 °C, with DMSO being the most effective. Attempts to lower the reaction temperature by using NMP and DMSO (Table 2, entries 6 and 8) as the solvent resulted in incomplete reaction after 3 h, so the most efficient reaction conditions for the intramolecular [4 + 2] cycloaddition of diene-tethered alkynyl bromide **1a** were found to be [IrCl(COD)]_2_ (2 mol %), dppe (4 mol %) in DMSO at 90 ºC.

**Scheme 3:**
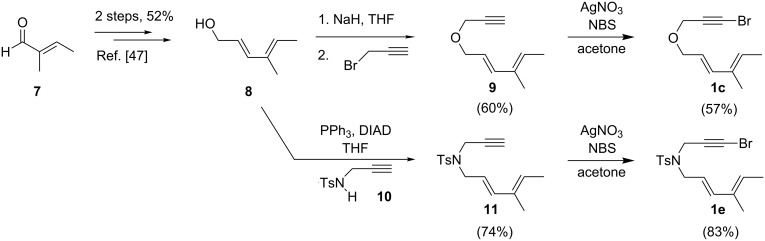
Synthesis of diene-tethered alkynyl halides **1c** and **1e**.

In order to illustrate the general applicability of this catalytic system, a variety of diene-tethered alkynyl halides were synthesized, including three previously unreported substrates **1c**, **1e**, and **1g** (Table 3). Alcohol **8** was prepared in two steps from tiglic aldehyde (**7**) as described by Gilbertson [47], and dienynes **9** and **11** could be synthesized from **8** (Scheme 3). Deprotonation of **8** with sodium hydride, followed by trapping with propargyl bromide provided **9** in 60% yield, and a Mitsunobu reaction between **8** and sulfonamide **10** (prepared as per reference [47]) provided **11** in 74% yield. Bromination of **9** and **11** provided diene-tethered alkynyl halides **1c** (57%) and **1e** (83%), respectively.

**Scheme 4:**
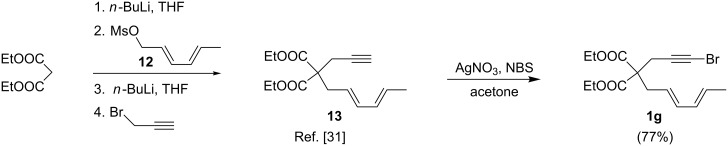
Synthesis of diene-tethered alkynyl halide **1g**.

Synthesis of substrate **1g** commenced with deprotonation of diethyl malonate, followed by addition of mesylate **12**, and subsequent deprotonation and addition of propargyl bromide as described by Gilbertson [31] to provide dieneyne **13** (Scheme 4). Diene **13** was then treated under the standard bromination conditions to provide **1g** in 77% yield.

Compounds **1a–1g** were then subjected to the optimized cycloaddition conditions (Table 3). In general, the Ir-catalyzed [4 + 2] cycloadditions occurred smoothly for all substrates, forming bicyclic products **2** in good yield (75–94%) (Table 3), and in each case a single stereoisomer was formed, with the H at the ring junction and the R group *anti* to each other. The stereochemistry of the cycloadducts was assigned by comparison with spectral data from previous work. This stereochemistry is consistent with that of previously reported intramolecular [4 + 2] cycloadditions in which the relative stereochemistry was assigned by X-ray diffraction analysis [32] or GOESY NMR [33]. For both alkynyl bromide **1a** and alkynyl chloride **1b** (Table 3, entries 1 and 2) the reaction went to completion quite quickly; however, a decreased yield was seen in the cycloaddition of **1b**. Attempts at the cycloaddition with the analogous alkynyl iodide provided a complex mixture of products. Changing R^1^ = H to R^1^ = Me (compare entries 1 and 3, Table 3) increased the reaction time slightly to 4 h, and a lower yield was seen for the cycloaddition of **1c**. A similar trend was seen for the nitrogen-tethered substrates (Table 3, entries 4 and 5) when changing R^1^ = H to R^1^ = Me, as the reaction time increased from 3 to 4 h and the yield decreased slightly. The all-carbon tethered substrates (Table 3, entries 6 and 7) required longer reaction times, but the cycloadditions occurred in good yields. Changing R = H to R = Me had little effect on the yield (Table 3, entries 6 and 7).

**Table 3:** [IrCl(cod)]_2_/dppe-catalyzed [4 + 2] cycloadditions of various alkynyl halides.

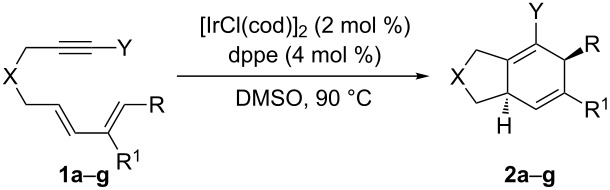								

Entry	Alkynyl halide	X	Y	R	R^1^	Time (h)	Cycloadduct	Yield^a^

1	**1a**	O	Br	Me	H	3	**2a**	94
2	**1b**	O	Cl	Me	H	3	**2b**	77
3	**1c**	O	Br	Me	Me	4	**2c**	75^b^
4	**1d**	NTs	Br	Me	H	4	**2d**	89
5	**1e**	NTs	Br	Me	Me	4	**2e**	77^c^
6	**1f**	C(CO_2_Et)_2_	Br	H	H	5	**2f**	80^d^
7	**1g**	C(CO_2_Et)_2_	Br	Me	H	5	**2g**	85

^a^Isolated yields after column chromatography. ^b^The aromatized product was also formed in trace amounts. ^c^Isolated yield after recrystallization from hexanes. ^d^The aromatized product was also formed in 6% yield.

**Scheme 5:**
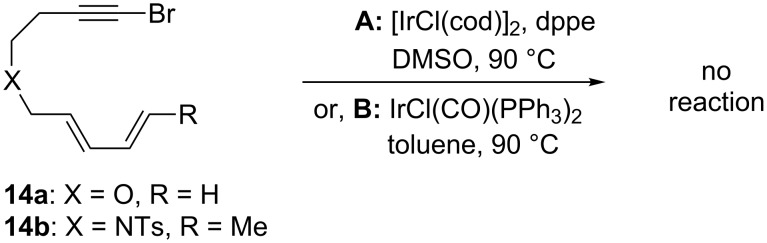
Unsuccessful cycloaddition attempts with substrates with a 4-atom tether.

Attempts at the cycloaddition reaction with substrates bearing a 4-atom tether were unsuccessful (Scheme 5). Two different substrates were synthesized and submitted to the optimized reaction conditions, but only the starting material was recovered. Changing the reaction conditions to the original system of Vaska’s complex in toluene (conditions **B**, Scheme 5) did not change this result. The previous Ir-catalyzed [4 + 2] cycloaddition work by Shibata and co-workers did not include any examples of similar substrates [26]; however, previous work in our group has shown that Rh-catalyzed [4 + 2] cycloadditions of this type of substrate occur in good yield [33].

These cycloadducts **2** are interesting due to the retention of the halogen in the cycloaddition reaction. This opens up the possibility of further coupling reactions to gain access to complex bicyclic molecules that would not be accessible directly by cycloaddition. Previous work has demonstrated this synthetic utility by the further functionalization of **2a** by Pd-catalyzed Suzuki and Heck coupling reactions [33]. In theory any of the cycloadducts **2** could undergo similar transformations, making them valuable intermediates that can be easily extended by metal-catalyzed coupling reactions.

## Conclusion

In conclusion, we have shown the first successful examples of Ir-catalyzed intramolecular [4 + 2] cycloadditions of alkynyl halides. The cycloadditions proceeded smoothly at 90 °C to afford the bicyclic products in good yields, and oxidative insertion of the iridium into the carbon–halide bond did not appear to be a problem. In addition to the known compounds synthesized, two previously unreported alkynyl halide substrates were synthesized, and were also found to undergo [4 + 2] cycloaddition under the reported conditions. The Ir-catalyzed reactions provided the cycloadducts in similar yields to the previous Rh-catalyzed reactions of alkynyl halides; however, the Ir-catalyzed reactions required higher reaction temperatures (90 versus 25 °C).

## Supporting Information

File 1 Experimental procedures for new compounds **1c**, **1e**, **1g**, and cycloadducts **2a–g**.

File 2 Copies of ^1^H and ^13^C NMR spectra for new compounds **1c**, **1e**, **1g**, and cycloadducts **2a–g**.
