# Supplementary material for: Iridium-catalyzed intramolecular [4 + 2] cycloadditions of alkynyl halides
Source: Beilstein J Org Chem. 2012 Oct 16;8:1765–70. doi: 10.3762/bjoc.8.201 (PMC3511010; doi:10.3762/bjoc.8.201)
Supplement: File 1 — Experimental procedures for new compounds 1c, 1e, 1g, and cycloadducts 2a–g. [file Beilstein_J_Org_Chem-08-1765-s001.pdf]

Supporting Information

for

Iridium-catalyzed intramolecular [4 + 2] cycloadditions of alkynyl halides

Andrew Tigchelaar and William Tam*

Address: Guelph-Waterloo Centre for Graduate Work in Chemistry and Biochemistry, Department of Chemistry, University of Guelph, Guelph, Ontario, Canada N1G 2W1

E-mail: William Tam – wtam@uoguelph.ca

* Corresponding author

Experimental procedures for new compounds 1c, 1e, 1g, and cycloadducts 2a–g.

Table of contents

General information S2
Alkynyl halide **1c** S2
Alkynyl halide **1e** S2
Alkynyl halide **1g** S3
Cycloadduct **2a** S4
Cycloadduct **2b** S4
Cycloadduct **2c** S4
Cycloadduct **2d** S5
Cycloadduct **2e** S5
Cycloadduct **2f** S6
Cycloadduct **2g** S6
References..................... S7

General Information: All reactions were carried out in an atmosphere of dry nitrogen or argon. ^{1}H and ^{13}C spectra were recorded on a 400 MHz spectrometer. Compounds **9** [1], **10** [1], **13** [2], **1a** [2], **1b** [2], **1d** [2], and **1f** [2] were all prepared as previously reported.

Alkynyl halide 1c (Scheme 3). In a round-bottomed flask, dienyne **9** (116.3 mg, 0.7742 mmol) was dissolved in acetone (4 mL). *N*-Bromosuccinimide (1.3 equiv, 182.5 mg, 1.014 mmol) and silver nitrate (0.2 equiv, 26.9 mg, 0.1584 mmol) were added to the reaction mixture and stirred for 3 h. The crude mixture was purified directly by column chromatography (EtOAc/hexanes 1:49) to provide **1c** (99.9 mg, 0.436 mmol, 57%) as a pale yellow oil. R_f 0.65 (EtOAc/hexanes 1:9); IR (neat, NaCl) 3035 (s), 2855 (s), 2213 (s), 1443 (s), 1087 (s), 968 (m), 926 (m), 850 (s); ^{1}H NMR ($CDCl_3$, 400 MHz) δ 6.29 (d, 1H, *J* = 15.6 Hz), 5.63 (dt, 2H, *J* = 15.4, 6.6 Hz), 4.15 (s, 2H), 4.10 (d, 2H, *J* = 6.5 Hz), 1.73 (s, 3H), 1.73 (d, 3H, *J* = 6.6 Hz); ^{13}C NMR (APT, $CDCl_3$, 100 MHz) δ 139.0, 133.8, 128.0, 121.1, 76.4, 70.7, 57.7, 45.8, 13.9, 12.0; HRMS (EI-TOF) calcd for $C_{10}H_{13}BrO$: *m/z* 228.0150, found *m/z* 228.0155.

Alkynyl halide 1e (Scheme 3). Sulfonamide **10** (1.3 equiv, 910.8 mg, 4.352 mmol) and triphenylphosphine (1.1 equiv, 1.0131 g, 3.863 mmol) were added to a round-bottomed flask and dissolved in THF (35 mL). Alcohol **8** (382.0 mg, 3.406 mmol) was added as a solution in THF (5 mL), and then DIAD (1.1 equiv, 0.74 mL, 3.758 mmol) was added dropwise by syringe. The reaction was stirred at rt overnight under nitrogen. The reaction was quenched with H_2O (40 mL), and diluted with ethyl acetate. The layers were separated, and then the aqueous layer was extracted three times with ethyl acetate. The combined organic layer was washed with deionized water, followed by saturated sodium chloride, then dried over magnesium sulfate, filtered, and concentrated by using rotary evaporation. The crude product **11** (766.0 mg, 2.524 mmol, 74% crude yield) was carried on unpurified to the next reaction. R_f 0.28 (EtOAc/hexanes 1:4). **1e** was synthesized following the same procedure as the synthesis of alkynyl bromide **1c**, by using dienyne

11 (333.3 mg, 1.098 mmol). The crude product was purified by column chromatography (EtOAc/hexanes 1:9) to provide **1e** (347.7 mg, 0.9094 mmol, 83%) as a clear oil. R_f 0.43 (EtOAc/hexanes 1:4); IR (neat, NaCl) 3039 (w), 2924 (s), 2256 (w), 2217 (w), 1649 (w), 1598 (w), 1448 (w), 1348 (s), 1161 (m), 1093 (m), 969 (m), 909 (m), 735 (s); ^{1}H NMR ($CDCl_3$, 400 MHz) δ 7.72 (d, 2H, *J* = 8.2 Hz), 7.31 (d, 2H, *J* = 8.1 Hz), 6.20 (d, 1H, *J* = 15.5 Hz), 5.57 (dd, 1H, *J* = 13.6, 6.7 Hz), 5.52 (dt, 1H, *J* = 15.6 Hz, 6.9 Hz), 4.05 (s, 2H), 3.82 (d, 2H, *J* = 7.0 Hz), 2.42 (s, 3H), 1.71 (d, 3H, *J* = 6.8 Hz), 1.67 (s, 3H); ^{13}C NMR (APT, $CDCl_3$, 100 MHz) δ 143.7, 140.0, 135.7, 133.6, 129.6, 128.4, 127.8, 119.0, 73.0, 49.0, 44.8, 36.8, 21.6, 13.9, 12.0; HRMS (ESI-Quadrupole TOF) calcd for $C_{17}H_{20}BrNO_2S$: [*m* + H]/*z* 382.0471, found [*m* + H]/*z* 382.0476.

Alkynyl halide 1g (Scheme 4): Following the same procedure as the synthesis of alkynyl bromide **1c**, by using diene-tethered terminal alkyne **14** (63.0 mg, 0.226 mmol). The crude product was purified directly by column chromatography (EtOAc/hexanes 1:39) to provide **1g** (59.9 mg, 0.174 mmol, 77%) as a pale yellow oil. R_f 0.35 (EtOAc/hexanes 1:9); IR (neat, NaCl) 3019 (m), 2981 (s), 2935 (s), 2873 (m), 1732 (s), 1445 (m), 1368 (m), 1284 (m), 1202 (s), 1096 (m), 990 (s), 913 (m), 735 (m); ^{1}H NMR ($CDCl_3$, 400 MHz) δ 6.09 (dd, 1H, *J* = 14.8, 10.4 Hz), 5.98 (dd, 1H, *J* = 14.9, 10.5 Hz), 5.63 (dq, 1H, *J* = 14.8, 6.8 Hz), 5.31 (dt, 1H, *J* = 14.8, 7.7 Hz), 4.19 (q, 4H, *J* = 7.1 Hz), 2.77 (m, 4H), 1.70 (d, 3H, *J* = 6.6 Hz), 1.25 (t, 6H, *J* = 7.1 Hz); ^{13}C NMR (APT, $CDCl_3$, 100 MHz) δ 169.7, 135.2, 131.0, 129.1, 123.5, 75.1, 61.7, 57.0, 41.2, 35.5, 23.9, 18.0, 14.1; HRMS (EI-TOF) calcd for $C_{16}H_{21}BrO_4$: *m*/*z* 356.0623, found *m*/*z* 356.0619 .

General procedure for the Ir-catalyzed [4 + 2] cycloaddition of 1a (Table 3, entry 1)

Alkynyl halide **1a** (1.0 equiv, 55.7 mg, 0.259 mmol) was added to an oven-dried screw-cap vial containing a stir bar. The vial was purged with nitrogen and brought into the glovebox. $[IrCl(cod)]_2$ (0.02 equiv, 3.4 mg, 0.0051 mmol) and dppe (0.04 equiv, 4.8 mg, 0.012 mmol) were added to a separate oven-dried vial, dissolved in DMSO (0.5 mL) and

stirred for 10 min. The Ir-catalyst mixture in DMSO was added to the vial containing **1a** by pipette, and the vial was rinsed with 0.8 mL DMSO and sealed. The reaction was stirred outside the glovebox for 3–5 h at 90 °C. The crude product was purified by column chromatography to yield the corresponding cycloadduct.

Cycloadduct 2a (Table 3, entry 1). The crude product was purified by column chromatography (EtOAc/hexanes 1:9) to provide cycloadduct **2a** (52.1 mg, 0.242 mmol, 94%) as a pale yellow oil. R_f 0.30 (EtOAc/hexanes 1:19); ^{1}H NMR ($CDCl_3$, 400 MHz) δ 5.69 (m, 2H), 4.62 (m, 2H), 4.23 (t, 1H, J = 7.1 Hz), 3.36 (dd, 1H, J = 11.4, 7.2 Hz), 3.26 (m, 1H), 3.12 (m, 1H), 1.31 (d, 3H, J = 7.3 Hz); ^{13}C NMR (APT, $CDCl_3$, 100 MHz) δ 138.1, 133.1, 120.8, 117.7, 72.4, 70.8, 43.9, 37.4, 21.5. Since this compound aromatized on standing no mass spectrum or elemental analysis was obtained (^{1}H and ^{13}C NMR spectra attached). Spectral data are consistent with those previously reported [2].

Cycloadduct 2b (Table 3, entry 2). Following the general cycloaddition procedure by using alkyne **1b** (25.7 mg, 0.151 mmol). The crude product was purified by column chromatography (EtOAc/hexanes 1:9) to provide cycloadduct **2b** (19.9 mg, 0.117 mmol, 77%) as a pale yellow oil. R_f 0.28 (EtOAc/hexanes 1:19); ^{1}H NMR ($CDCl_3$, 400 MHz) δ 5.68 (s, 2H), 4.41 (m, 2H), 4.19 (m, 1H), 3.32 (m, 2H), 3.07 (m, 1H), 1.29 (d, 3H, J = 7.3 Hz); ^{13}C NMR (APT, $CDCl_3$, 100 MHz) δ 134.6, 133.3, 125.5, 120.8, 72.2, 68.7, 43.4, 36.3, 19.8. Since this compound aromatized on standing no mass spectrum or elemental analysis was obtained (^{1}H and ^{13}C NMR spectra attached). Spectral data are consistent with those previously reported [2].

Cycloadduct 2c (Table 3, entry 3). Following the general cycloaddition procedure by using alkyne **1c** (52.3 mg, 0.228 mmol). The crude mixture was purified by column chromatography (EtOAc/hexanes 1:19) to provide **2c** (39.2 mg, 0.171 mmol, 75%) as a pale yellow oil. R_f 0.40 (EtOAc/hexanes 1:9); IR (neat, NaCl) 2926 (s), 2856 (s), 1463

(m), 1188 (m), 1055 (s), 904 (m); ^{1}H NMR ($CDCl_3$ 400 MHz) δ 5.43 (s, 1H), 4.31 (m, 2H), 4.18 (t, 1H, *J* = 6.4 Hz), 3.30 (m, 1H), 3.24 (m, 1H), 2.98 (m, 1H), 1.77 (s, 3H), 1.34 (d, 3H, *J* = 7.2 Hz); ^{13}C NMR (APT, $CDCl_3$, 100 MHz) δ 138.3, 137.9, 118.6, 116.5, 72.7, 70.7, 44.4, 41.4, 22.2, 19.8; HRMS (EI-TOF) calcd for $C_{10}H_{13}BrO$: *m*/*z* 228.0150, found *m*/*z* 228.0154.

Cycloadduct 2d (Table 3, entry 4). Following the general cycloaddition procedure by using alkyne **1d** (29.0 mg, 0.0787 mmol). The crude product was purified by column chromatography (EtOAc/hexanes 1:9) to provide cycloadduct **2d** (25.9 mg, 0.0703 mmol, 89%) as a yellow solid. Mp 85–86 °C; R_f 0.19 (EtOAc/hexanes 1:9); IR (neat, NaCl) 3424 (s), 2933 (s), 2857 (s), 1696 (m), 1454 (m), 1345 (s), 1160 (s), 1047 (m), 954 (w), 855 (m); ^{1}H NMR ($CDCl_3$, 400 MHz) δ 7.72 (d, 2H, *J* = 8.3 Hz), 7.34 (d, 2H, *J* = 8.0 Hz), 5.61 (br s, 2H), 4.00 (dq, 1H, *J* = 14.7, 1.6 Hz), 3.89 (dd, 1H, *J* = 8.8, 7.5 Hz), 3.79 (dt, 1H, *J* = 14.6, 1.9 Hz), 3.15 (m, 1H), 2.95 (m, 1H), 2.69 (dd, 1H, *J* = 11.6, 8.8 Hz), 2.43 (s, 3H), 1.21 (d, 3H, *J* = 7.3 Hz); ^{13}C NMR ($CDCl_3$, 100 MHz) δ 143.7, 134.5, 133.6, 132.7, 129.8, 127.6, 120.9, 120.3, 52.8, 52.1, 41.9, 37.2, 21.5, 21.4; HRMS (ESI-Quadrupole TOF) calcd for $C_{16}H_{18}BrNO_2S$: [*m*+H]/*z* 368.0320, found [*m*+H]/*z* 368.0324. Spectral data are consistent with those previously reported [2].

Cycloadduct 2e (Table 3, entry 5). Following the general cycloaddition procedure by using alkyne **1e** (99.3 mg, 0.260 mmol). The crude mixture was flushed through a plug of silica with EtOAc to provide the impure product. Recrystallization from hot hexanes provided **2e** (76.4 mg, 0.200 mmol, 77%) as a white solid. Mp 142–143 °C; R_f 0.29 (EtOAc/hexanes 1:9); IR (neat, NaCl) 2959 (m), 2929 (s), 2858 (m), 1732 (s), 1460 (m), 1123 (w), 1073 (w), 854 (s); ^{1}H NMR ($CDCl_3$, 400 MHz) δ 7.73 (d, 2H, *J* = 8.2 Hz), 7.33 (d, 2H, *J* = 8.0 Hz), 5.34 (s, 1H), 3.99-3.94 (m, 3H), 3.10 (m, 1H), 2.88 (m, 1H), 2.43 (s, 3H), 1.55 (s, 3H), 1.24 (d, 3H, *J* = 7.2 Hz); ^{13}C NMR (APT, $CDCl_3$, 100 MHz) δ 143.6,

138.2, 134.3, 133.8, 129.8, 127.6, 121.0, 116.6, 53.2, 52.1, 42.5, 41.3, 22.1, 21.6, 19.7; HRMS (ESI-Quadrupole TOF) calcd for $C_{17}H_{20}BrNO_2S$: [*m*+H]/*z* 382.0471, found [*m*+H]/*z* 382.0478.

Cycloadduct 2f (Table 3, entry 6). Following the general cycloaddition procedure by using alkyne **1f** (65.0 mg, 0.1894 mmol). The crude mixture was purified by column chromatography (EtOAc/hexanes 1:9) to provide **2f** (52.1 mg, 0.152 mmol, 80%) as a pale yellow oil. R_f 0.30 (EtOAc/hexanes 1:9); IR (neat, NaCl) 3033 (m), 2981 (s), 2828 (m), 1747 (s), 1465 (m), 1431 (m), 1299 (s), 1154 (s), 1096 (s), 1065 (s), 1008 (m), 961 (m); ^{1}H NMR ($CDCl_3$, 400 MHz) δ 5.76 (m, 1H), 5.62 (m, 1H), 4.20 (m, 4H), 3.05 (m, 5H), 2.65 (m, 1H), 1.76 (m, 1H), 1.25 (m, 6H); ^{13}C NMR ($CDCl_3$, 100 MHz) δ 171.8, 171.2, 137.7, 125.7, 125.1, 112.0, 61.7, 61.6, 57.1, 42.0, 39.9, 39.2, 37.0, 14.0 (2). Anal. calcd for $C_{15}H_{19}BrO_4$: C, 52.49; H, 5.58; found: C, 52.11; H, 5.75. Spectral data are consistent with those previously reported [2].

Cycloadduct 2g (Table 3, entry 7). Following the general cycloaddition procedure by using alkyne **1g** (40.5 mg, 0.118 mmol). The crude mixture was purified by column chromatography (EtOAc/hexanes 1:9) to provide **2g** (34.6 mg, 0.101 mmol, 85%) as a pale yellow oil. R_f 0.40 (EtOAc/hexanes 1:9); IR (neat, NaCl) 2981 (s), 2933 (m), 2873 (w), 1732 (s), 1446 (m), 1255 (s), 1188 (m), 1071 (m), 860 (m), 737 (m); ^{1}H NMR ($CDCl_3$, 400 MHz) δ 5.68 (d, 1H, *J* = 9.7 Hz), 5.55 (d, 1H, *J* = 9.8 Hz), 4.21 (m, 4H), 3.04 (m, 4H), 2.64 (m, 1H), 1.87 (t, 1H, *J* = 12.5 Hz), 1.25 (m, 9H); ^{13}C NMR (APT, $CDCl_3$, 100 MHz) δ 171.9, 171.3, 137.9, 131.4, 123.9, 119.8, 61.8, 61.7, 57.7, 42.5, 39.9, 39.6, 37.7, 22.0, 14.1, 14.0; HRMS (EI-TOF) calcd for $C_{16}H_{21}BrO_4$: *m*/*z* 356.0623, found *m*/*z* 356.0617.

References

1. DeBoef, B.; Counts, W.R.; Gilbertson, S.R. *J. Org. Chem.* **2007**, *72*, 799-804. doi:10.1021/jo0620462
2. Yoo, W.J.; Allen, A.; Villeneuve, K.; Tam, W. *Org. Lett.* **2005**, *7*, 5853-5856. doi:10.1021/ol052412o
