# Supplementary material for: Iridium-catalyzed intramolecular [4 + 2] cycloadditions of alkynyl halides
Source: Beilstein J Org Chem. 2012 Oct 16;8:1765–70. doi: 10.3762/bjoc.8.201 (PMC3511010; doi:10.3762/bjoc.8.201)
Supplement: File 2 — Copies of 1H and 13C NMR spectra for new compounds 1c, 1e, 1g, and cycloadducts 2a–g. [file Beilstein_J_Org_Chem-08-1765-s002.pdf]

Supporting Information

for

Iridium-catalyzed intramolecular [4 + 2] cycloadditions of alkynyl halides

Andrew Tigchelaar and William Tam*

Address: Guelph-Waterloo Centre for Graduate Work in Chemistry and Biochemistry, Department of Chemistry, University of Guelph, Guelph, Ontario, Canada N1G 2W1

E-mail: William Tam – wtam@uoguelph.ca

* Corresponding author

Copies of ^{1}H and ^{13}C NMR spectra for new compounds **1c**, **1e**, **1g**, and cycloadducts **2a–g**

Table of contents

^{1}H and ^{13}C spectra of alkynyl halide **1c** S2
^{1}H and ^{13}C spectra of alkynyl halide **1e** S3
^{1}H and ^{13}C spectra of alkynyl halide **1g** S4
^{1}H and ^{13}C spectra of cycloadduct **2a** S5
^{1}H and ^{13}C spectra of cycloadduct **2b** S6
^{1}H and ^{13}C spectra of cycloadduct **2c** S7
^{1}H and ^{13}C spectra of cycloadduct **2d** S8
^{1}H and ^{13}C spectra of cycloadduct **2e** S9
^{1}H and ^{13}C spectra of cycloadduct **2f** S10
^{1}H and ^{13}C spectra of cycloadduct **2g** S11

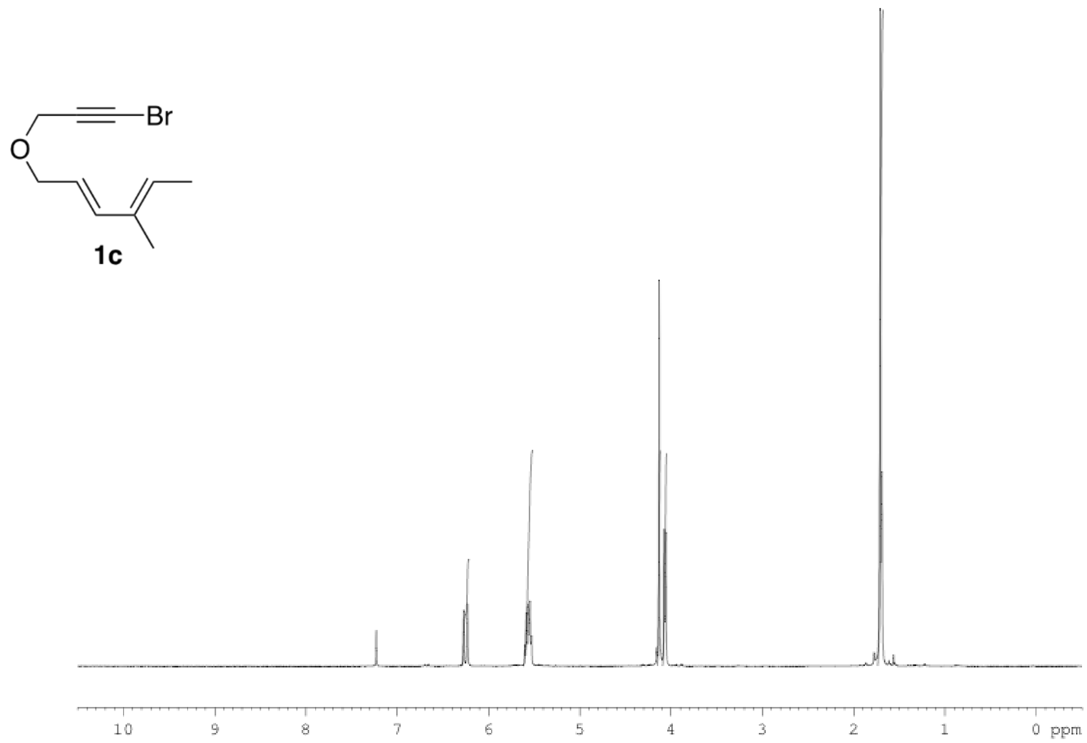

Figure 1: 400 MHz ^{1}H NMR spectrum in $CDCl_3$.

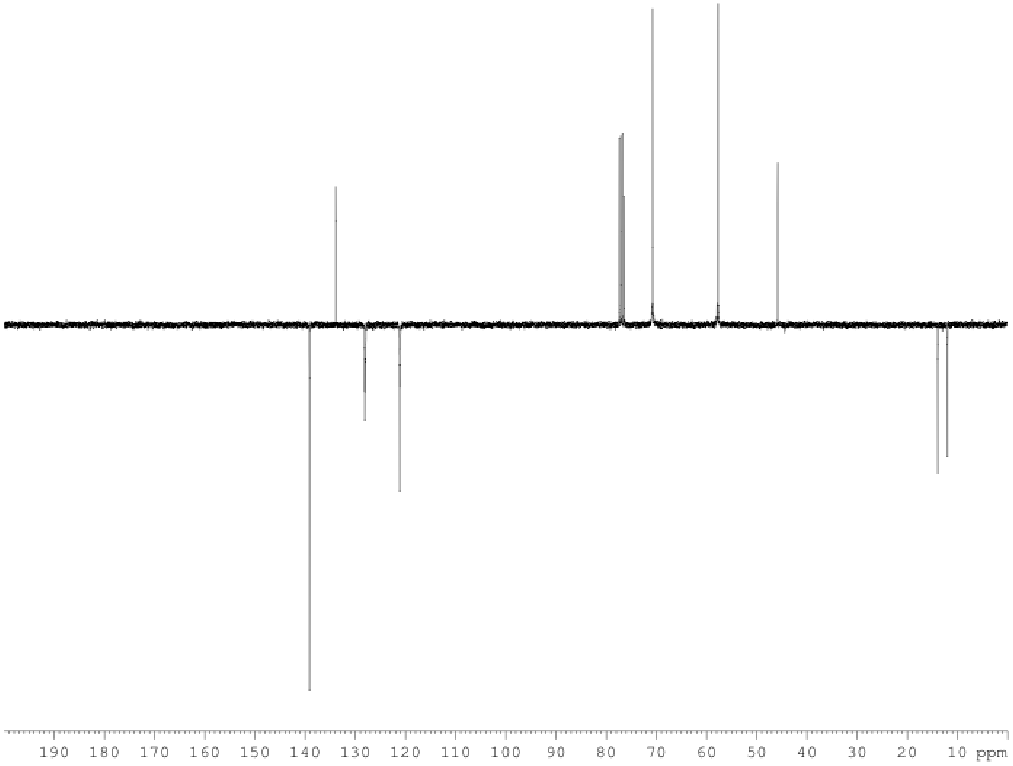

Figure 2: 100 MHz ^{13}C NMR spectrum in $CDCl_3$.

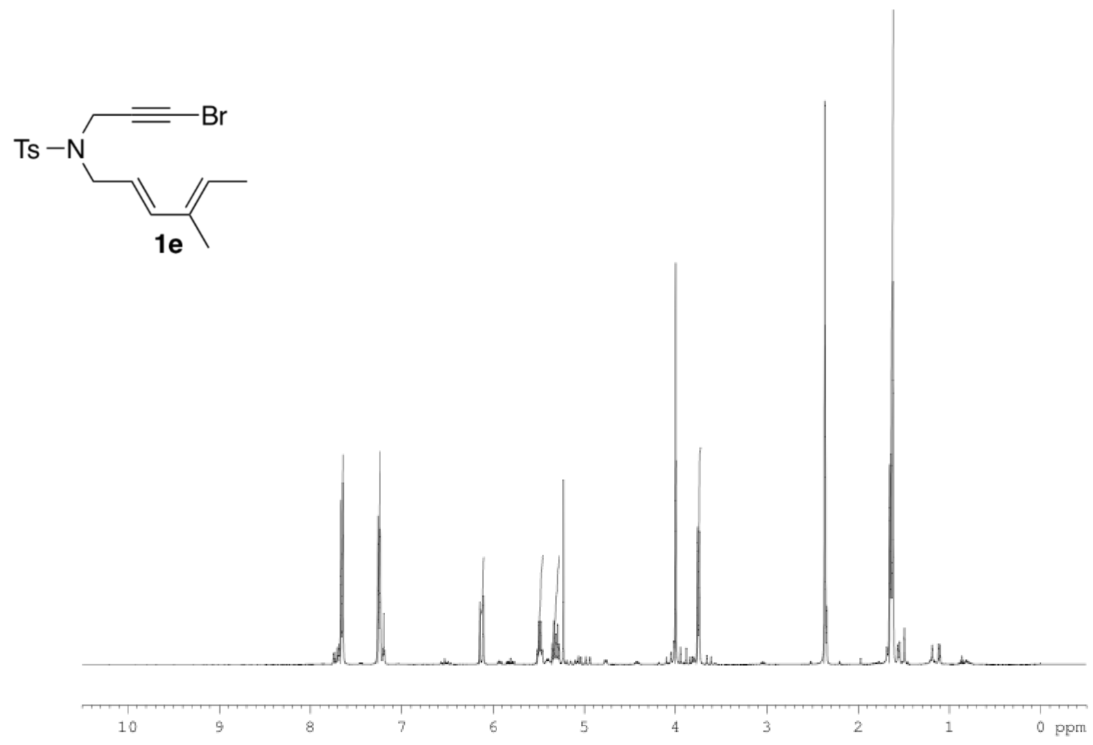

Figure 3: 400 MHz ^{1}H NMR spectrum in $CDCl_3$.

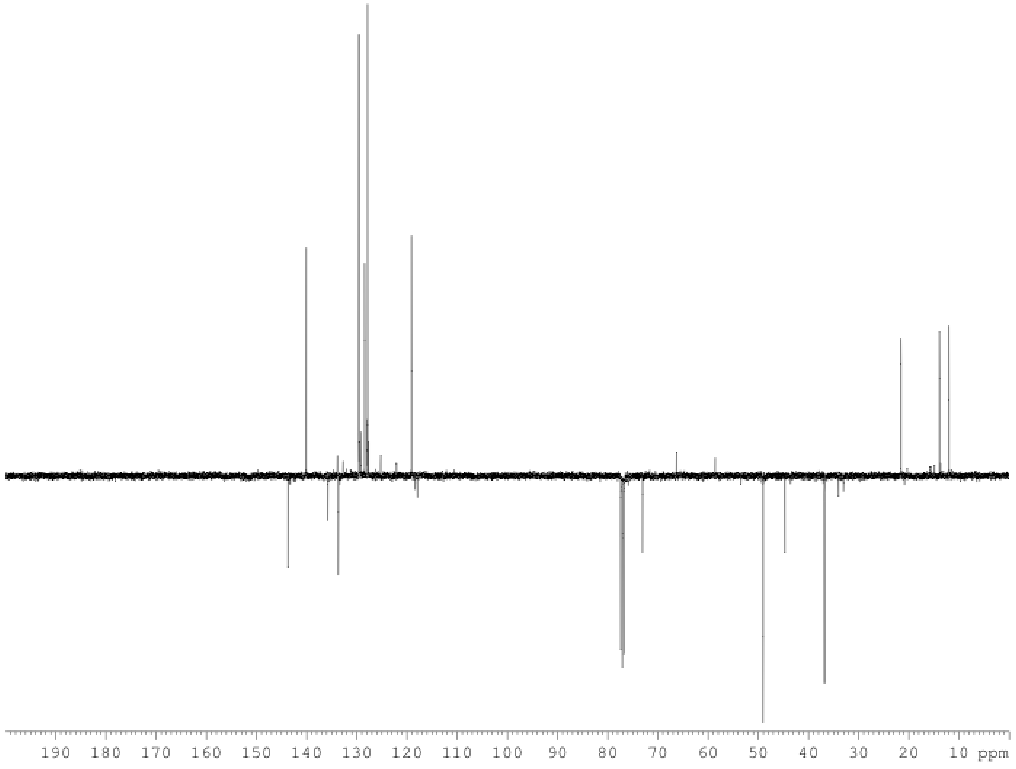

Figure 4: 100 MHz ^{13}C NMR spectrum in $CDCl_3$.

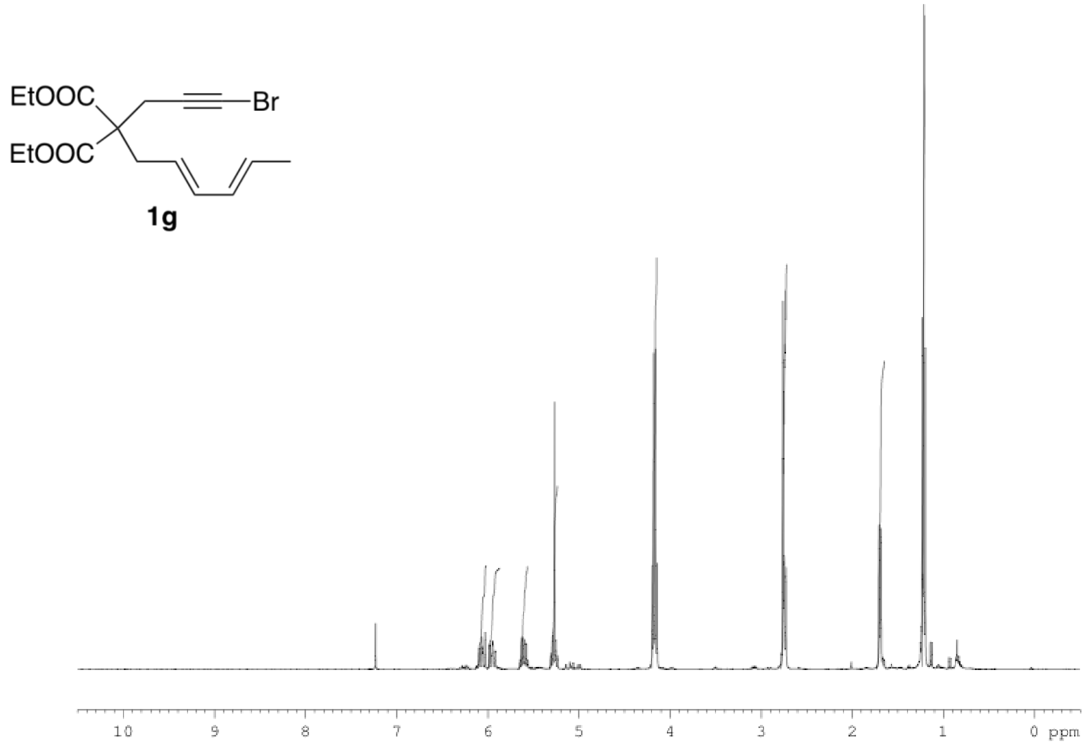

Figure 5: 400 MHz ^{1}H NMR spectrum in $CDCl_3$.

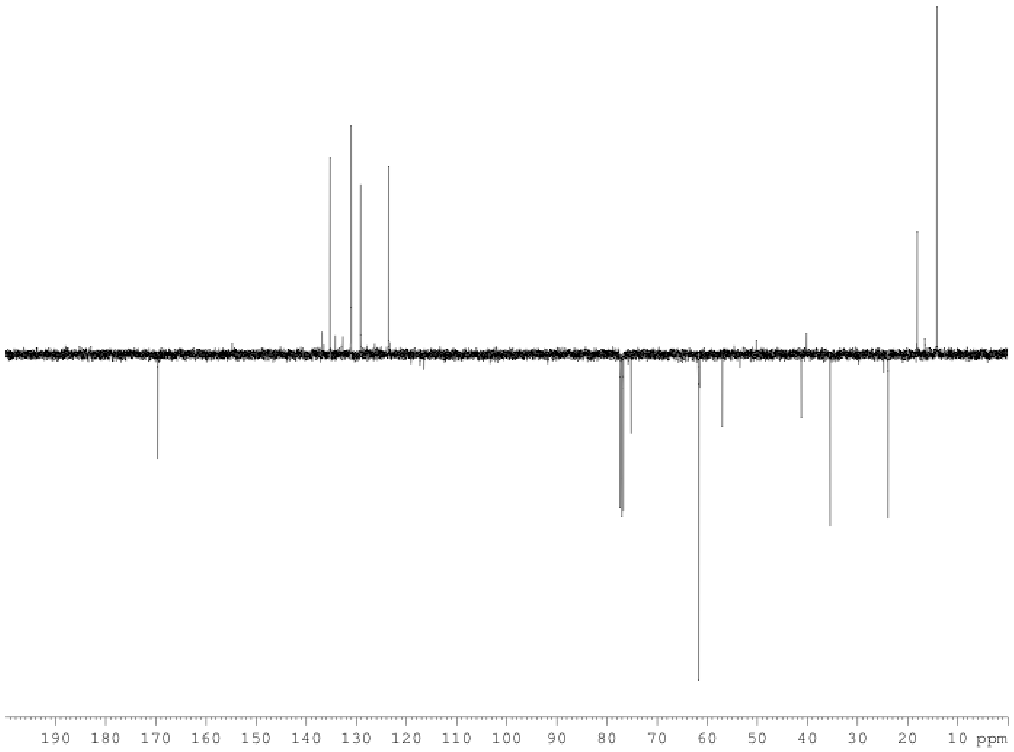

Figure 6: 100 MHz ^{13}C NMR spectrum in $CDCl_3$.

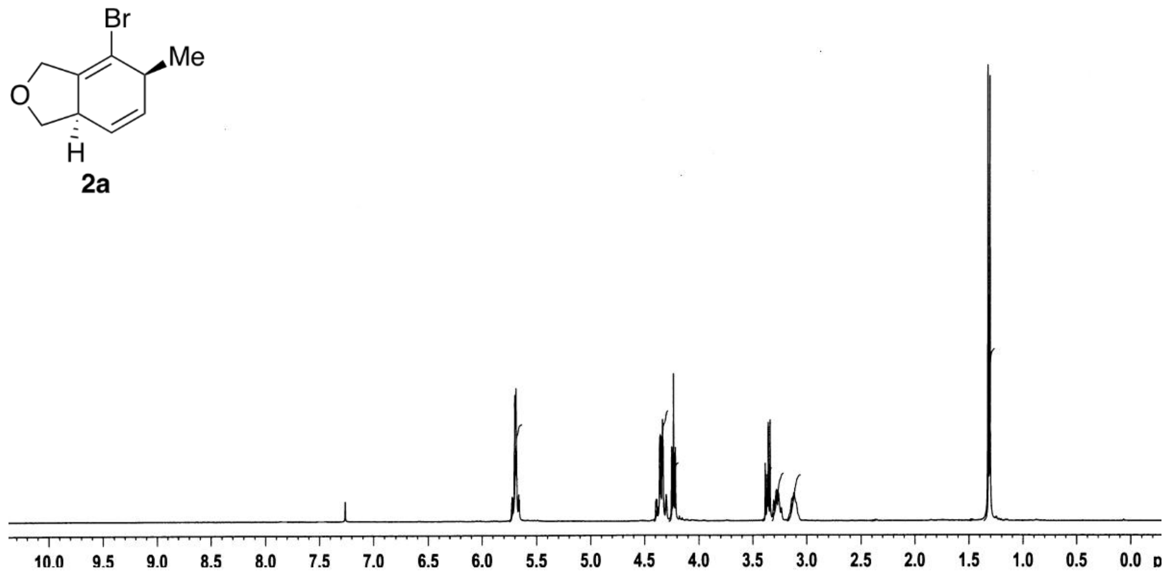

Figure 7: 400 MHz ^{1}H NMR spectrum in $CDCl_3$.

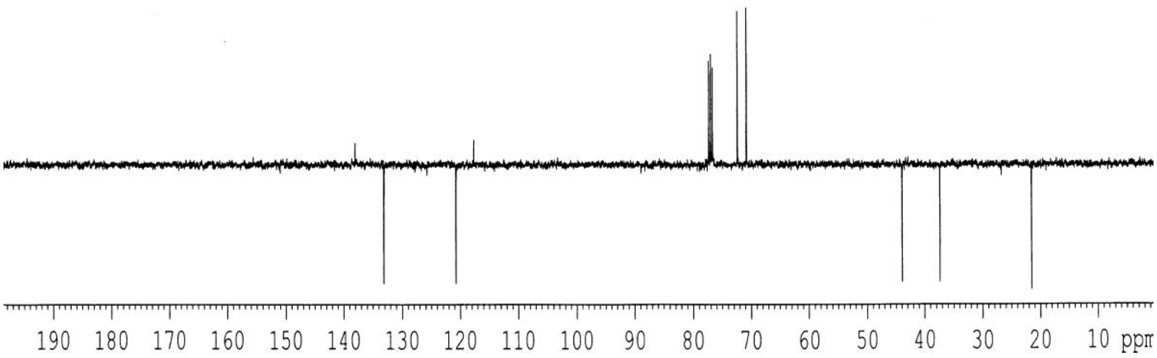

Figure 8: 100 MHz ^{13}C NMR spectrum in $CDCl_3$.

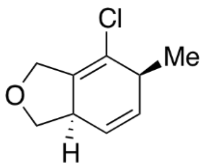

2b

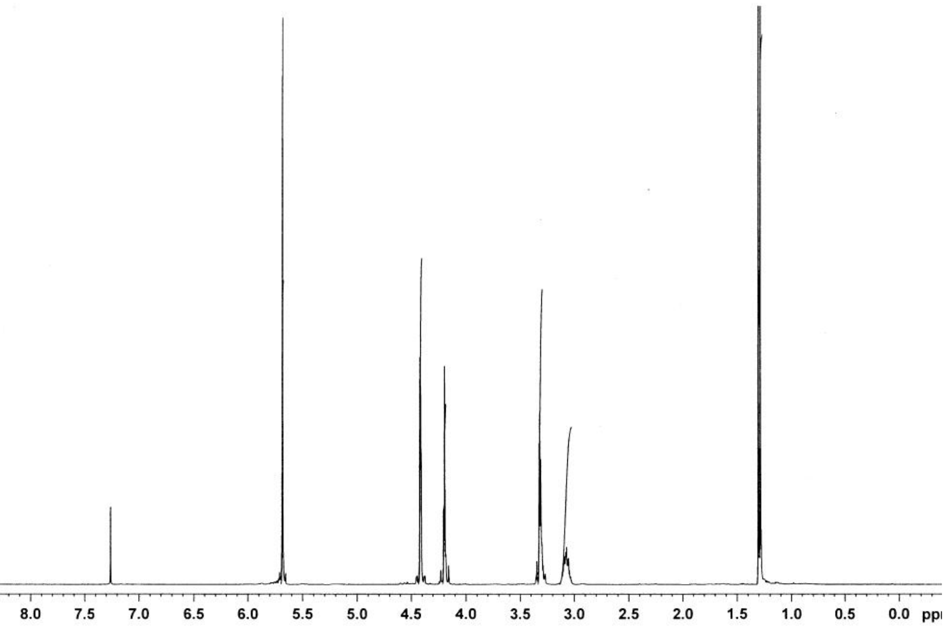

Figure 9: 400 MHz ^{1}H NMR spectrum in $CDCl_3$.

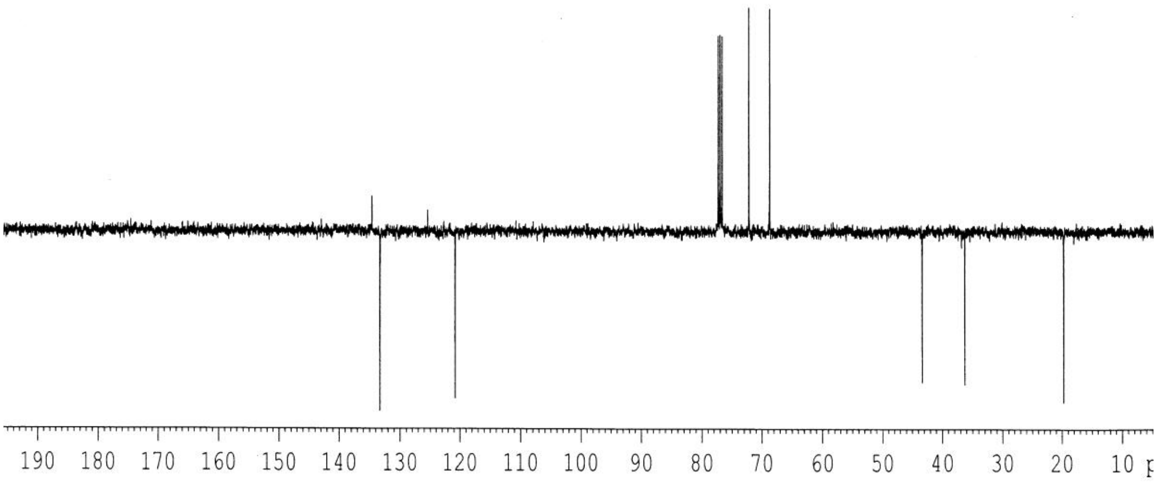

Figure 10: 100 MHz ^{13}C NMR spectrum in $CDCl_3$.

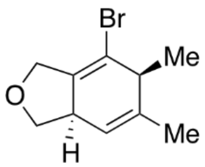

2c

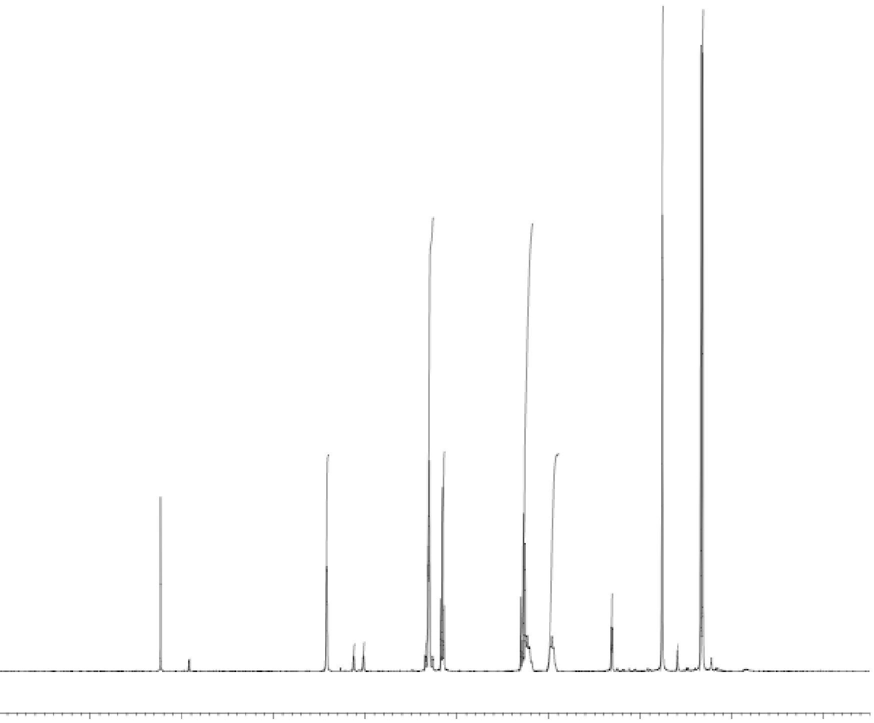

Figure 11: 400 MHz ^{1}H NMR spectrum in $CDCl_3$.

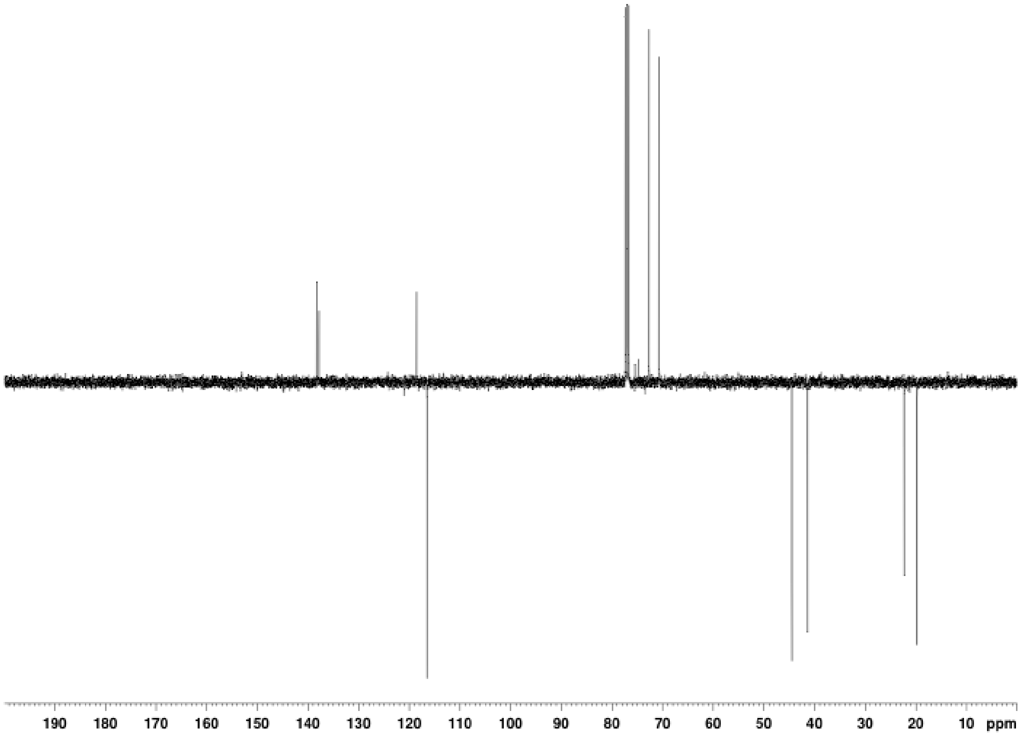

Figure 12: 100 MHz ^{13}C NMR spectrum in $CDCl_3$.

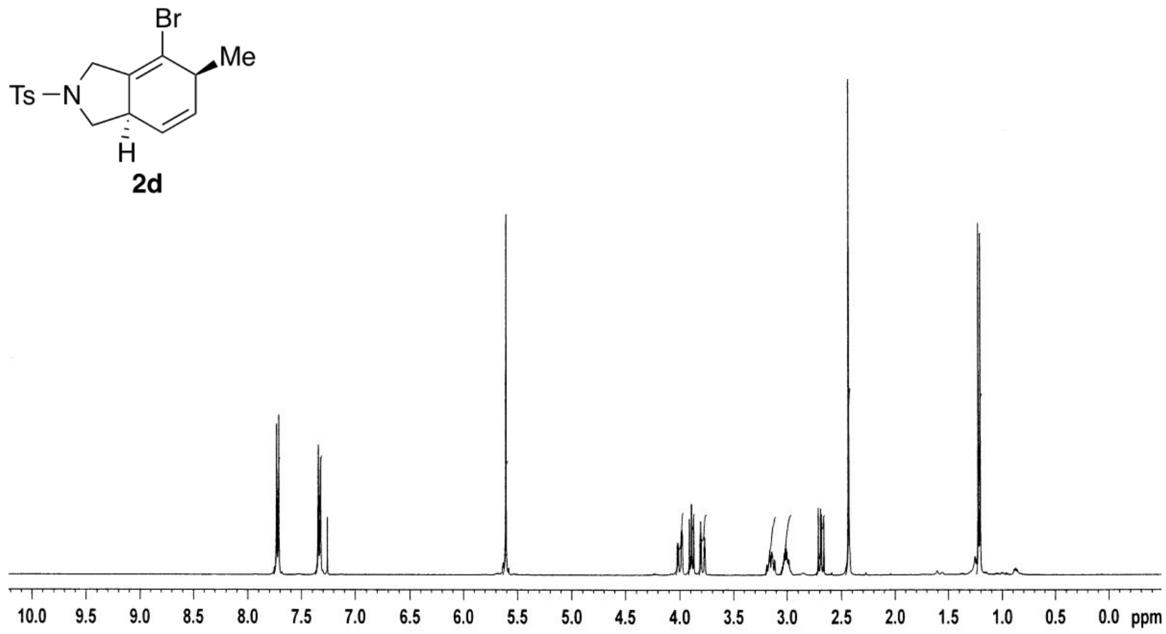

Figure 13: 400 MHz ^{1}H NMR spectrum in $CDCl_3$.

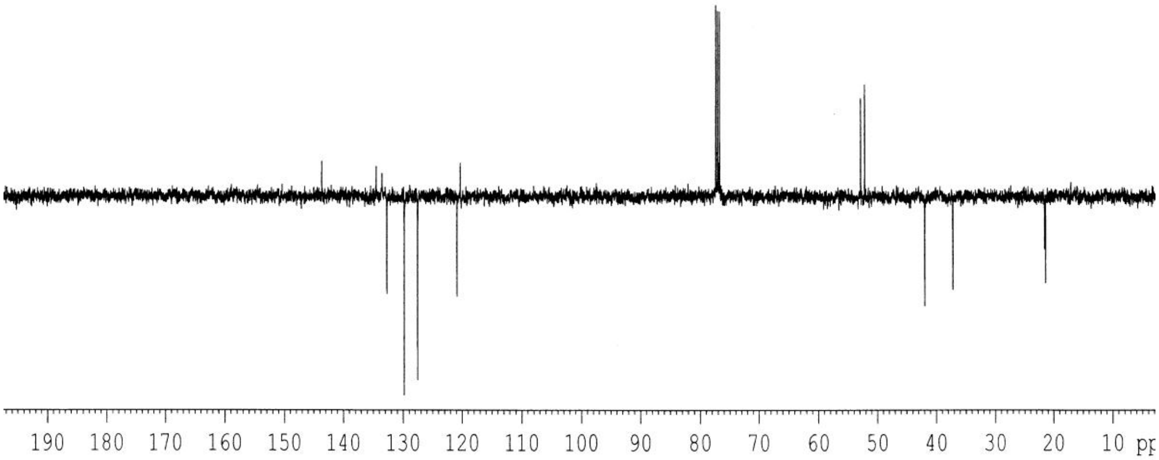

Figure 14: 100 MHz ^{13}C NMR spectrum in $CDCl_3$.

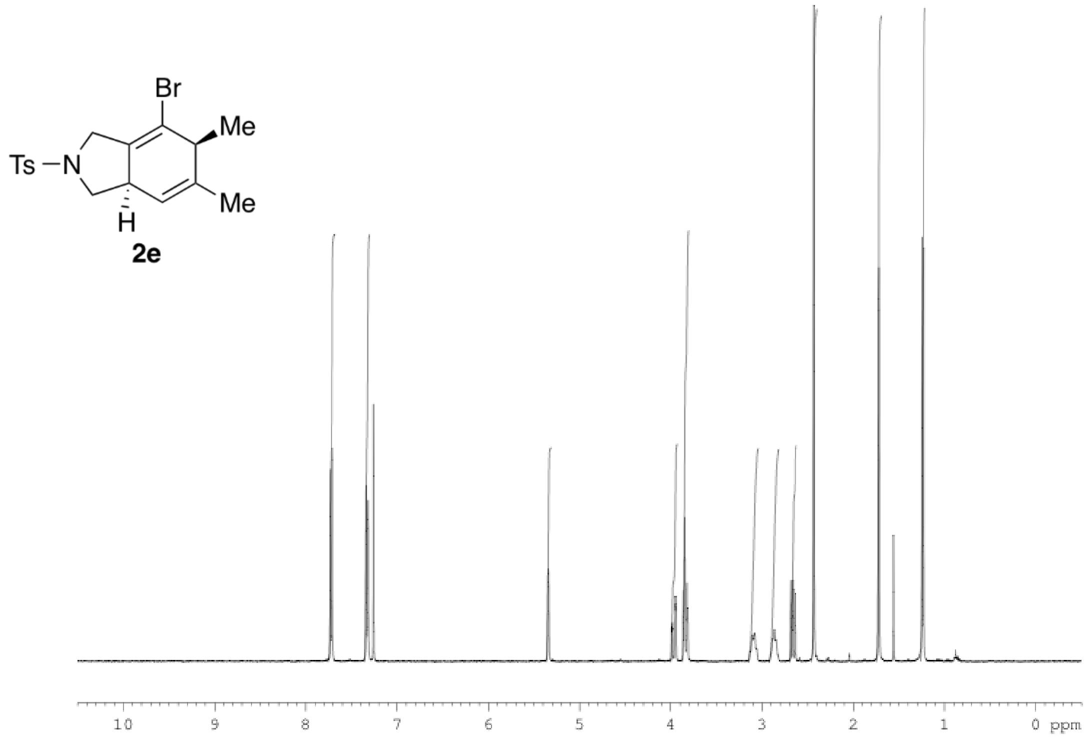

Figure 15: 400 MHz ^{1}H NMR spectrum in $CDCl_{3.}$

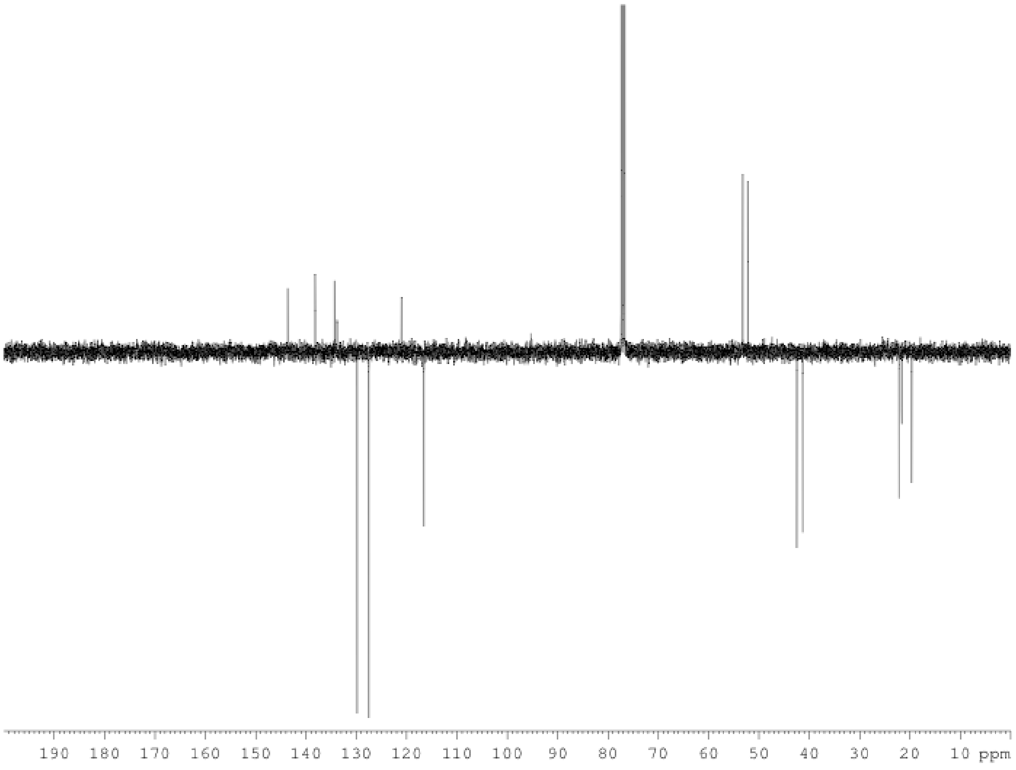

Figure 16: 100 MHz ^{13}C NMR spectrum in $CDCl_{3.}$

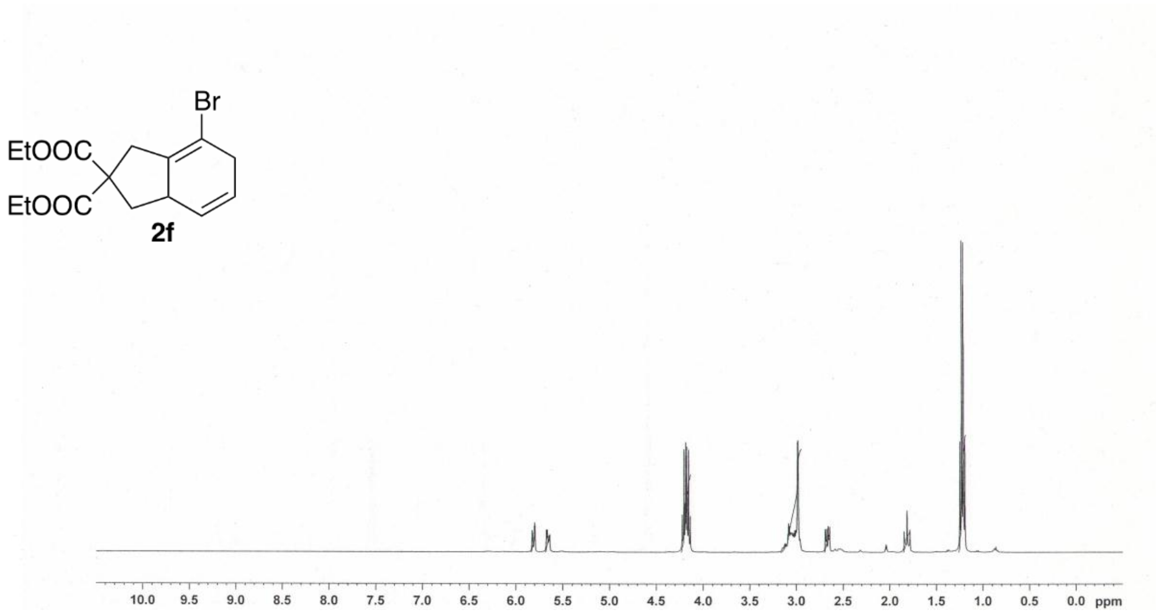

Figure 17: 400 MHz ^{1}H NMR spectrum in $CDCl_3$.

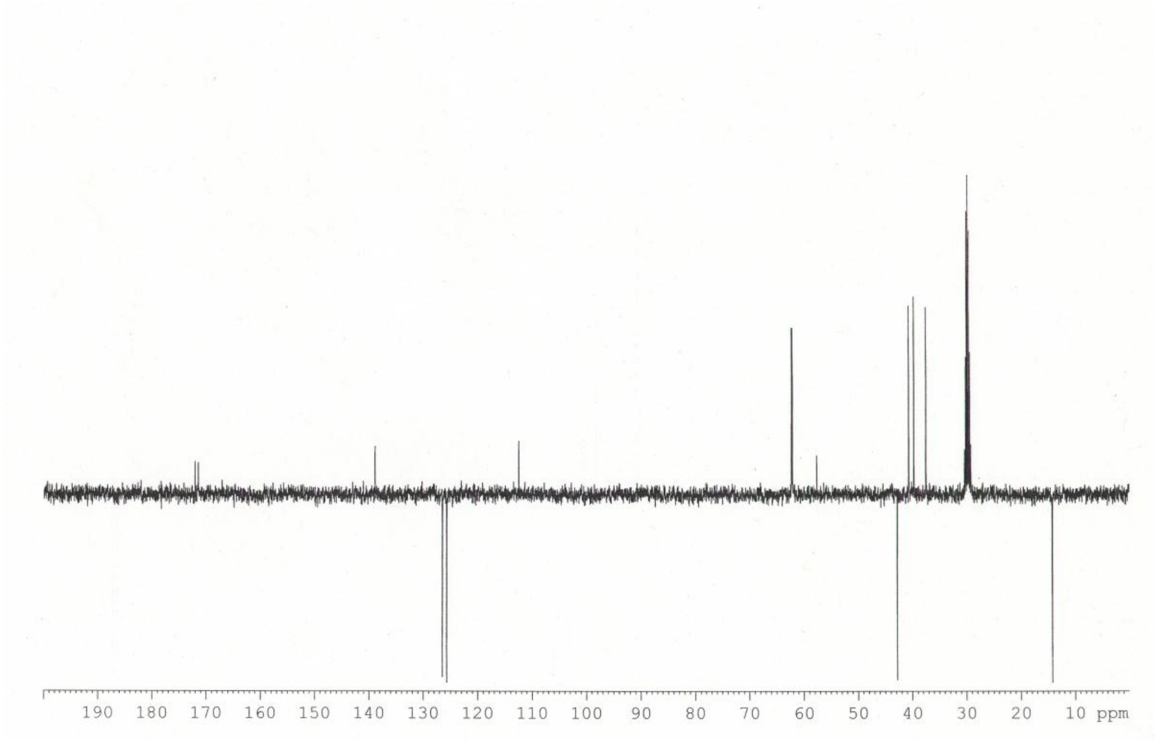

Figure 18: 100 MHz ^{13}C NMR spectrum in $CDCl_3$.

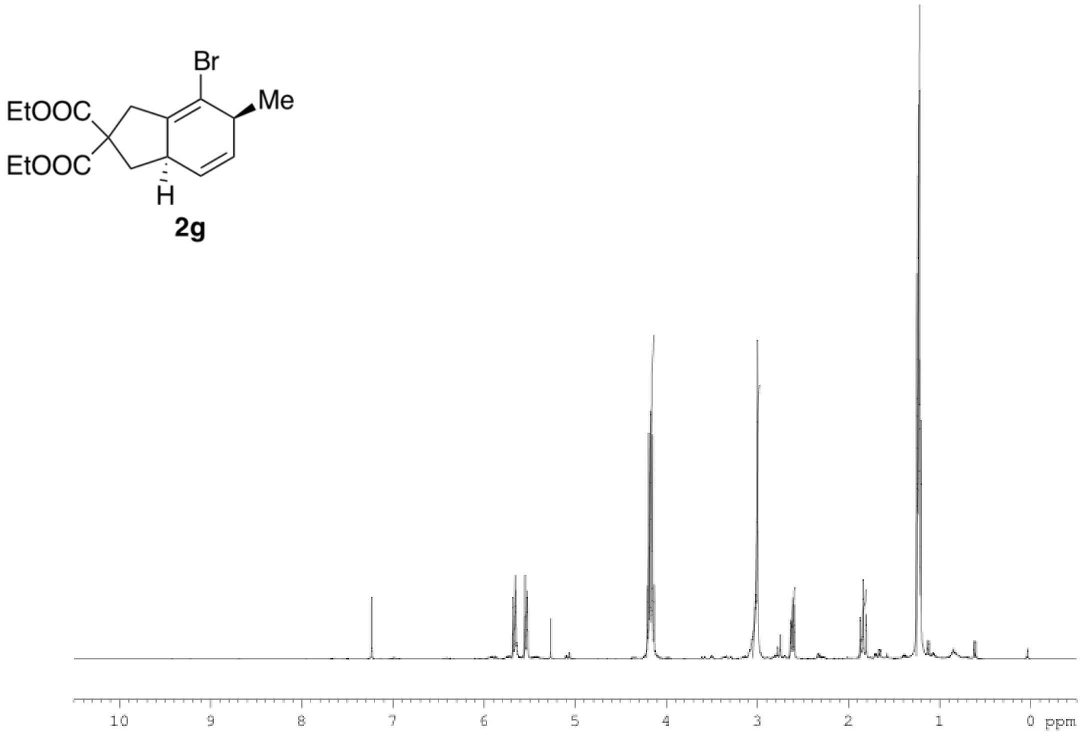

Figure 19: 400 MHz ^{1}H NMR spectrum in $CDCl_3$.

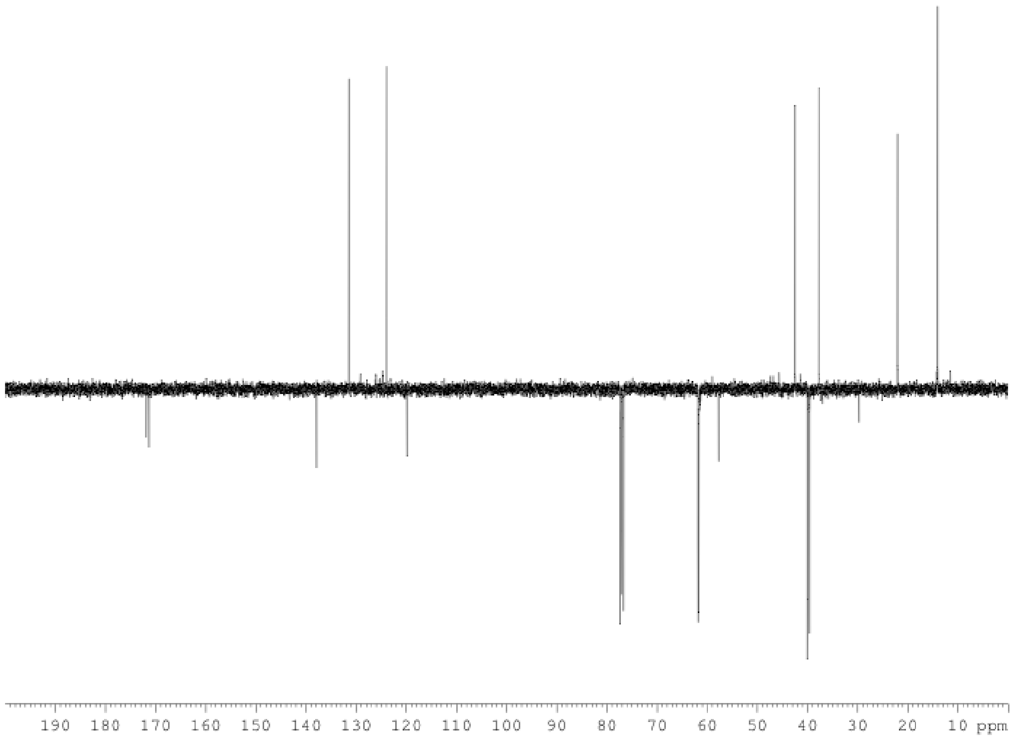

Figure 20: 100 MHz ^{13}C NMR spectrum in $CDCl_3$.
